# Effect of Platelet-Rich Plasma on CCl_4_-Induced Chronic Liver Injury in Male Rats

**DOI:** 10.1155/2014/932930

**Published:** 2014-02-23

**Authors:** Zahra Hesami, Akram Jamshidzadeh, Maryam Ayatollahi, Bita Geramizadeh, Omid Farshad, Akbar Vahdati

**Affiliations:** ^1^Department of Biology, Science and Research Branch, Islamic Azad University, Fars, Iran; ^2^Pharmaceutical Sciences Research Center, Shiraz University of Medical Sciences, Shiraz, Iran; ^3^Transplant Research Center, Shiraz University of Medical Sciences, Shiraz, Iran; ^4^International Branch, Shiraz University of Medical Sciences, Shiraz, Iran

## Abstract

Platelet-rich plasma (PRP) has been of great concern to the scientists and doctors who are involved in wound healing and regenerative medicine which focuses on repairing and replacing damaged cells and tissues. Growth factors of platelet-rich plasma are cost-effective, available, and is more stable than recombinant human growth factors. Given these valuable properties, we decided to assess the effect of PRP on CCl_4_-induced hepatotoxicity on rats. The rats received CCl_4_ (1 mL/kg, i.p. 1 : 1 in olive oil) twice per week for 8 weeks. Five weeks after CCl_4_ injection, the rats also received PRP (0.5 mL/kg, s.c.) two days a week for three weeks. Twenty-four hours after last CCl_4_ injection, the animals bled and their livers dissected for biochemical and histopathological studies. Blood analysis was performed to evaluate enzyme activity. The results showed that PRP itself was not toxic for liver and could protect the liver from CCl_4_-induced histological damages and attenuated oxidative stress by increase in glutathione content and decrease in lipid peroxidative marker of liver tissue. The results of the present study lend support to our beliefs in hepatoprotective effects of PRP.

## 1. Introduction

Liver is considered the key organ in the metabolism, detoxification, and secretory functions in the body, and its disorders are numerous with no effective remedies; however, the search for new medicines is still ongoing. It is a hematopoietic organ in the fetal period, and mature hepatocytes produce thrombopoietin, which can stimulate platelet production in bone marrow. However, few studies have investigated the relationship between hematic components, that is, platelets and liver regeneration [1–4].

The platelet-rich plasma (PRP) used in tissue regeneration serves as a developing area for clinicians and researchers. It is well known that platelets have a thrombotic effect. Platelets contain not only proteins needed for hemostasis but also many growth factors such as transforming growth factor (TGF), platelet-derived growth factor (PDGF), vascular endothelial growth factor (VEGF), epidermal growth factor (EGF), and insulin-like growth factor (IGF) [5].

Some study has been reported; platelets accumulate in the liver under some kinds of pathologic conditions, like ischemia/reperfusion injury [6–8], liver cirrhosis [9], cholestatic liver [10], and viral hepatitis [11].

Many in vitro studies demonstrated that platelets contain several growth factors which may theoretically contribute to the process of liver regeneration [12, 13]. However, there are few studies on the role of platelets in liver regeneration in rats that failed to identify a correlation between platelets and liver regeneration [14, 15].

Carbon tetrachloride (CCl_4_) is a widely used chemical for experimental induction of fatty liver and liver fibrosis in animals [16]. Its biotransformation produces hepatotoxic metabolites, the highly reactive trichloromethyl-free radical, subsequently converted to the peroxytrichloromethyl radical [17]. Based on the findings of different studies in this field, the present study was undertaken to investigate hepatoprotective activity of PRP on the liver injury induced by CCl_4_ in experimental animal model. 

## 2. Materials and Methods

### 2.1. Chemicals

CCl_4_, calcium gluconate, sodium dodecyl sulfate, ethylenediaminetetraacetic acid (EDTA), 5,5′-dithiobis-(2-nitrobenzoic acid) or DTNB, tris, thiobarbituric acid (TBA), and trichloroacetic acid were from Sigma Chemical Company, Germany. All other chemicals were of highest quality available in the market.

### 2.2. Preparing Platelet-Rich Plasma

Fourteen female rats (170–200 g) were selected from the Laboratory Animals Research Center in Shiraz University of Medical Sciences. The rats were maintained under controlled temperature and 12 hours light/12 hours dark conditions for one week before the start of the experiments. They were allowed to feed on standard laboratory chaw and tap water ad libitum. The research protocol complied with the guidelines for animal care of our institution.

The rats were anesthetized with ether, followed by blood collection from the rats via open chest cardiac puncture. About 100 mL of blood was collected from them after killing. The blood was then mixed with sodium citrate (3.8%) (9 parts of blood to 1 part of sodium citrate) anticoagulant solution. Then, the blood was centrifuged at 1000 rpm for 15 min at 20°C for separation of platelet rich plasma. Also, the plasma was centrifuged at 3000 rpm for 10 min at 20°C to obtain platelet pellet. The platelet concentrate dissolved in phosphate buffer saline (PBS), pooled and incubated at room temperature for 30 min on a rotating platform to eliminate platelet agglomerates. Platelets were counted using Sysmex KX-21 (Japan), resulting in a platelet number of 679 × 10^3^/*μ*L [18]. Afterwards, autologous thrombin was prepared as per Lucarelli et al.'s method [19]. At this step, 330 *μ*L of calcium gluconate (100 mg/mL) was added to 10 mL of plasma and 1 mL of thrombin preparation to 4 mL of platelet concentrate and incubated for 1 h at room temperature to facilitate growth factors release. The platelet secretion was centrifuged at 4000 rpm for 5 min to reduce the presence of platelet membrane fragments. The supernatant was filtered with a 0.22 *μ*m pore filter, divided into aliquots, and frozen at −80°C for subsequent use [20]. The protein concentration in the filtrates was determined by Bradford method (150 mg/mL) [21].

### 2.3. Study Design

As designed, the 24 male Wistar rats (250–300 g) were randomly divided into 4 groups, each consisting of 6 animals. Group I received olive oil (0.5 mL/kg, i.p., *n* = 6) as normal control; group II received CCl_4_ (1 mL/kg body weight as a 1 : 1 mixture with olive oil i.p., *n* = 6) twice per week for 8 weeks; group III received PRP (0.5 mL/kg 1 : 1 in PBS, s.c. *n* = 6) two days a week for three weeks; and group IV received the CCl_4_ (1 mL/kg body weight as a 1 : 1 mixture with olive oil i.p.) twice per week for 8 weeks. Five weeks after CCl_4_ injection, the rats received PRP (0.5 mL/kg 1 : 1 in PBS s.c. *n* = 6) two days a week for three weeks. Twenty-four hours after CCl_4_ injection, the animals were anaesthetized by sodium thiopental injection (50 mg/kg) and their blood samples collected from the vena cava. Then, the respective sera were separated for subsequent use of different enzyme measurements. The rats were then decapitated and their livers carefully dissected and cleaned of extraneous tissues, and parts of the liver tissue were immediately transferred to 10% formalin for histopathological assessments.

### 2.4. Histopathological Studies

Part of the liver was removed from the animals and the tissue fixed in 10% formalin for at least 24 hours. Then, the paraffin sections were prepared (by Automatic tissue processor, Autotechnique) and cut into 5 *μ*m thick sections by a rotary microtom. The sections then were stained with Haematoxylin-Eosin dye and studied for histopathological changes, that is, necrosis, fatty changes, ballooning degeneration, and Inflammation. Histological damage is scored as follows: 0: absent; +: mild; ++: moderate; and +++: severe.

### 2.5. Measurement of ALT, AST, and Albumin in Serum

Biocon standard kits and DAX-48 autoanalyzer were used to measure alanine aminotransferase (ALT), aspartate aminotransferase (AST), and albumin (ALB) activities in serum, according to Wilkinson et al.'s and Bessay et al.'s method [22, 23].

### 2.6. Determination of Lipid Peroxidation

The lipid peroxidation extent was assessed by measuring the amount of thiobarbituric acid-reactive substances (TBARs). In Brief, 500 mg of liver tissue gently minced in 4.5 mL of 0.25 M sucrose. The minced tissues gently homogenized and then centrifuged at 2000 rpm for 30 min. Afterwards, 0.1 mL of the supernatant was treated with a buffer containing 0.75 mL of thiobarbituric acid (0.8%, w/v), 0.75 mL of 20% acetic acid (pH = 3.5), and 0.1 mL of sodium dodecyl sulfate (8.1%, w/v). The solution was mixed up with 2 mL of distilled water and heated in a boiling water bath for 60 min. The absorbance then was measured at 532 nm by a Beckman DU-7 spectrophotometer [24].

### 2.7. GSH Determination

Glutathione reductase 5,50-dithiobis-2 nitrobenzoic acid (DTNB) recycling procedure [25] was used to determine the reduced glutathione. In brief, 100 mg of liver tissues was homogenized in a buffer containing EDTA (0.2 M) to obtain 4% (w/v) whole homogenate. Then, 1.5 mL of the suspension was taken and mixed with a buffer containing 2.5 mL distilled water and 0.5 mL of 50% TCA. The mixture then was centrifuged at 3000 rpm for 15 min and 1 mL of the supernatant mixed with 1 mL of Tris buffer (0.4 M, pH = 8.9) and 0.1 mL of DTNB (0.01 M). The absorbance was measured after 5 min at 412 nm using a Beckman DU-7 spectrophotometer [26].

### 2.8. Statistical Analysis

The data were analyzed by student's Tukey test and one-way ANOVA, followed by Graph pad Prism 5. The difference between the control and experimental groups was considered significant at *P* ≤ 0.05.

## 3. Results

**Figure 1:**
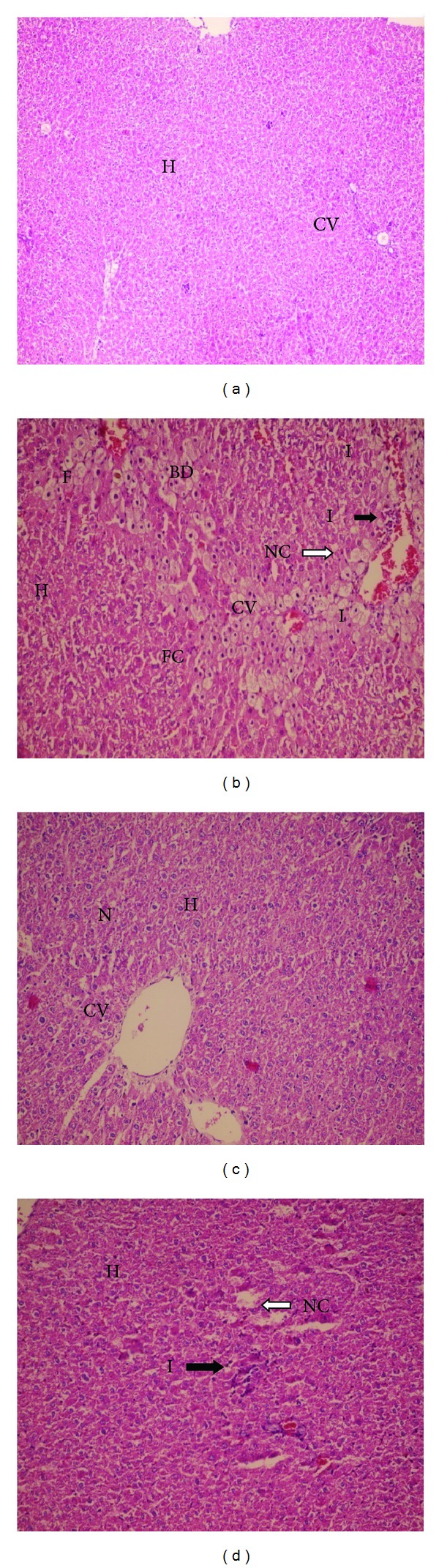
Effect of the platelet-rich plasma (PRP) on histopathological changes induced by CCl_4_ in rats. (a) (H&E ×100) liver section of normal rats showing normal hepatocytes with prominent nucleus, cytoplasm, and central vein; (b) (H&E ×250) liver sections of CCl_4_-treated (0.5 mL/kg i.p.) rats showing fatty chain, necrosis, and infiltration of inflammatory cells; (c) (H&E ×250) liver sections of the rats treated with PRP (0.5 mL/kg s.c.) showing well-brought out central vein hepatocytes with well-preserved cytoplasm and normal hepatocytes with prominent nucleus; (d) (H&E ×250) liver sections of the rats treated with CCl_4_ + PRP (0.5 mL/kg + 0.5 mL/kg s.c.) showing normal architecture of hepatocytes and mild infiltration of inflammatory cells. H: hepatocyte, CV: central vein, N: nucleus, F: foamy macrophage cells, FC: fatty chain, NC: necrosis, I: infiltration of inflammatory cells, and BD: ballooning degeneration.

Histopathological studies revealed that CCl_4_ imposed focal necrosis, fatty changes, ballooning degeneration, and infiltration of lymphocytes around the central veins (Figure 1(b); Table 1). Necrosis, which is a more severe form of injury, was markedly prevented by treatment with PRP (Figure 1(d)) and demonstrated a normal appearance, except for mild inflammation (+ in Table 1) in pericentral hepatocytes to the vein and mild necrosis (+ in Table 1). In the PRP groups that received only PRP there was no significant toxicity, which shows that PRP did not induce hepatotoxicity (Figure 1(c)).

**Figure 2:**
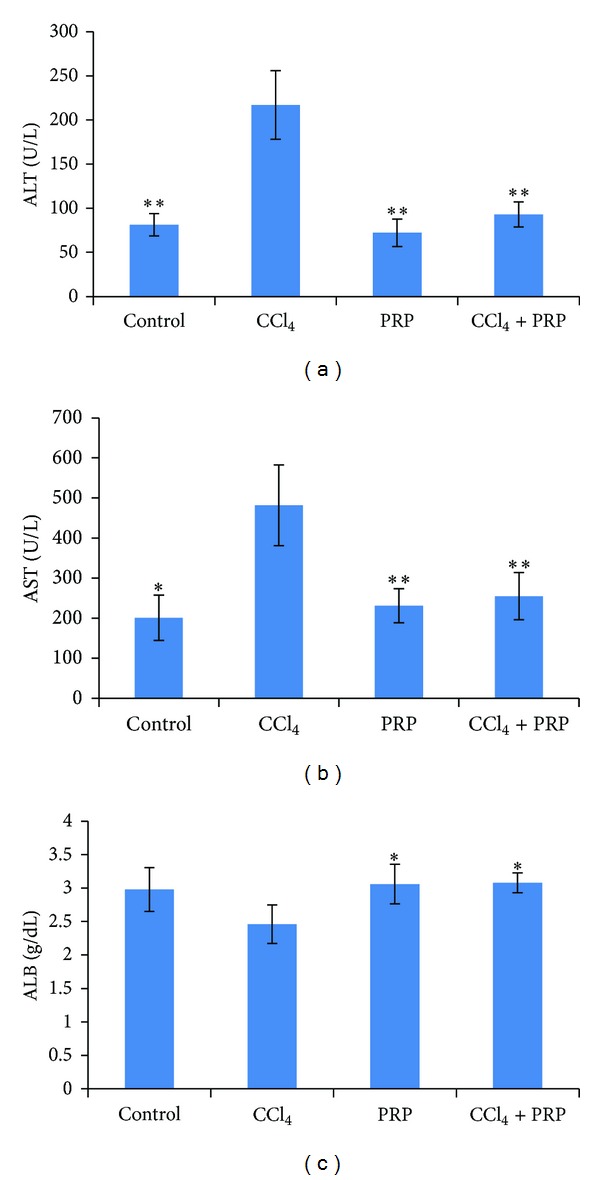
Effect of platelet rich plasma (PRP) on rat hepatic enzymes and albumin levels changed by CCl_4_. (a) ALT, (b) AST, (c) albumin. Rats were injected CCL_4_ with doses (0.5 mL/kg i.p. 1 : 1 in olive oil) twice per week for 8 weeks. Five weeks after CCl_4_ injection rats received PRP (0.5 mL/kg s.c.) 2 days a week for 3 weeks. Values are mean ± SD of 6 rats per group. *Significantly different from CCl_4_-treated group (*P* ≤ 0.05). **Significantly different from CCl_4_-treated group (*P* ≤ 0.01).

Results of enzyme activity analysis are presented in Figure 2. Administration of CCl_4_ to rats caused a significant elevation in serum ALT and AST activities after 8 weeks. Albumin (Figure 2(c)) did not show significant changes in CCl_4_-treated group, compared to the control group. Treatment of rats with 0.5 mg/kg i.p. of the PRP markedly prevented CCl_4_-induced elevation of serum ALT and AST (Figures 2(a) and 2(b)).

As shown in Table 2, the liver's lipid peroxidation was significantly increased in the CCl_4_ group when compared with the controls (*P* ≤ 0.01) and PRP ameliorated CCl_4_-induced increases of MDA concentration (*P* ≤ 0.01). These findings indicate that the oxidative stress in the liver was effectively decreased when treated with PRP.

Glutathione (GSH) is measured as an index of antioxidant status of liver. There was a significant increase of GSH content in the PRP groups, compared to the group that received CCl_4_ alone (Table 2).

## 4. Discussion

Carbon tetrachloride-induced hepatic injury is commonly used as an experimental method for the study of hepatoprotective effects of drugs or medicinal plants extracts, by in vivo and in vitro techniques [27, 28].

CCl_4_ is believed to be metabolized by microsomal CYP450 in the liver to a highly reactive trichloromethyl-free radical (^*∙*^CCl3) which can start a chain of reactive free radical formation resulting in peroxidation of lipids and damage to the proteins and components of the cells leading to cell lyses [29, 30].

Effect of platelets on liver regeneration was not addressed till the beginning of the 21st century. There are some reported studies in which platelets were shown to promote liver regeneration [27]. The following study was conducted to determine the role of platelets in liver regeneration using a thrombocytosis model in mice after 90% partial hepatectomy [28]. The entire disrupted platelets and the soluble fraction were with significant proliferative effects, whereas the membrane fraction had no significant effect. Studies indicate that the direct contact between platelets and hepatocytes could spark the release of soluble factors from the platelets such as IGF-1 and HGF; IGF-1 is contained in human platelet as the most important mediator for liver regeneration, which had a proliferative effect on them [29]. The findings in the other experiment demonstrate that exogenous platelets also enhance liver regeneration [30]. Meanwhile, growth factors, like vascular insulin-like growth factor I (IGF-I), endothelial growth factor (VEGF), and hepatocyte growth factor (HGF), contribute to hepatocyte proliferation that induced by platelet [31]. The growth factors stimulate onset of hepatocyte mitosis, which ultimately promote liver regeneration, especially in humans, since it was reported that human platelets do not contain a significant amount of HGF [32]. Most reports lend support to a decrease in platelet count associated with the severity of liver injury [33–35]. Carbon tetrachloride (CCl_4_) is a chemical agent used for experimental promotion of fatty liver and liver fibrosis in animals [16]. The present study was undertaken to investigate of the hepatoprotective activity of PRP against CCl_4_-induced damage in rat.

Lipid peroxidation is among the actual causes of CCl_4_-induced liver injury [36, 37] and is mediated by the free radical derivatives of CCl_4_. CCl_3_ radicals produced in reactions by animals microsomes liver exposed to CCl_4_ were assumed to attack the membrane lipid in endoplasmic reticulum of hepatocyte. When they attacked the membrane lipid in hepatocyte endoplasmic reticulum, malondialdehyde (MDA) emerged promptly [38]. The rise of MDA levels in the liver is indicative of an enhanced peroxidation that causes tissue damage and breakdown of the antioxidant defense mechanisms and thus inhibits the formation of superabundant free radicals [39]. In the present study, PRP administration caused a significant decrease in MDA levels, compared to the CCl_4_-treated rat, suggesting that PRP could protect against CCl_4_-induced lipid peroxidation in rats.

Unlike the toxic consequences of CCl_4_ metabolism through the CYP_2_E_1_ pathway, the detoxification pathway involves GSH conjugation of trichloromethyl-free radicals [40]. A reduced level of GSH is crucial in the detoxification of the reactive toxic metabolites of CCl_4_; liver necrosis is initiated when reserves of GSH are remarkably depleted [41]. In the present study, the hepatic content of GSH was found to be decreased significantly in CCl_4_ intoxicated rats, compared to the controls. Table 2 shows that PRP treatment significantly inhibited the CCl_4_-induced decrease of hepatic GSH content. When CCl_4_ is administered to rats, the actions of aspartate aminotransferase (AST) and alanine aminotransferase (ALT) in rat plasma rise remarkably with necrosis and lipid accumulation of hepatocyte [42]. Both enzymes are indicators of liver injury. ALT is more sensitive to acute liver injury test, whereas AST is more sensitive to chronic injury [43]. The present study showed that PRP treatment significantly improved levels of ALT, AST, and albumin after CCl_4_ administration (Figures 2(a), 2(b), and 2(c)). The most important protein synthesized by the liver is serum albumin whose main function is to regulate the colloidal osmotic pressure of blood and it reflects the extent of functioning liver cell mass. However, measuring of albumin can provide information to identify chronic injury among the experimental rats [44].

The biochemical observations are supported by the histopathological examination of rats' livers. As a result of hepatotoxicity of CCl_4_, significant regenerative cellular proliferation occurs to compensate for the necrotic or damaged tissue. The Histopathological result (Figure 1) shows severe necrosis in central vein of the rats treated with CCl_4_ compared to the control group that showed +++ degree (Table 1). This result is in accordance with other results [16] and shows fatty changes (+ grade in Table 1), compared to the control group.

We showed that fatty changes in PRP + CCl_4_ groups decrease (0 degree, Table 1), compared to CCl_4_ rat models (Figure 1(b)). The platelets are stimulated by a lot of motivation like infection, inflammation, and injury. Platelets have modulatory effects on inflammatory cell responses [45]. It seems that the histological changes in CCl_4_ group, in addition to necrosis, have a foci apoptotic lesion in their livers (Figure 1(b)). The grade of necrotic foci in the liver reduced from +++ in CCl_4_ to + in PRP treatment groups (Table 1).

There is evidence showing platelets to be effective in antifibrosis [46, 47], antiapoptosis [48], and liver regeneration [29]. Platelet therapy can open a new horizon to develop novel strategies for the treatments of liver diseases.

The results presented in this study indicated that the treatment of rats with the PRP 5 weeks after CCl_4_-induced toxicity leads to the reduction of hepatotoxicity. The hepatoprotective effects of PRP may be due to inhibited lipid peroxidation and effective recovery of the antioxidative defense system and has a remarkable effect on signal transduction. Overall, platelet-rich plasma can be used as a complementary procedure to decrease the destructive effects of hepatotoxicants.

## Figures and Tables

**Table 1:** Effect of the platelet-rich plasma (PRP) on histopathological liver damages induced by CCl_4_ in rats.

Groups	Ballooning Degeneration	Fatty change	Hepatocyte necrosis	Inflammation	Others
Control	0	0	0	0	—
CCl_4 _	++	+	+++	+	Many foamy macrophages and old necrosis
PRP	0	0	0	0	—
PRP + CCl_4 _	0	0	+	+	—

0: absent; +: mild; ++: moderate; +++: severe.

Rats were injected (i.p.) CCL_4_ with doses (0.5 mL/kg 1 : 1 in olive oil) twice per week for 8 weeks. Five weeks after CCl_4_ injection, rats received PRP (0.5 mL/kg 1 : 1 in PBS, s.c.). The PRP (0.5 mL/kg 1 : 1 in PBS, s.c.) alone did not increase the levels of the enzymes. Values are mean ± SD of 6 rats per group.

**Table 2:** Effects of platelet-rich plasma (PRP) on GSH and TBARs levels of the liver damaged by CCl_4_ in rats.

Groups	GSH (nmol/g liver)	TBARs (nmol/g liver)
Control	0.35454 ± 0.035*	0.7 ± 0.057**
CCL_4_ (0.5 mL/kg)	0.18727 ± 0.016	4.96 ± 0.84
PRP (0.5 mL/kg)	0.33187 ± 0.058*	0.962 ± 0.23**
CCL_4 _+ PRP	0.28774 ± 0.034*	0.981 ± 0.035**

GSH: reduced glutathione; TBARs: thiobarbituric acid-reactive substances.

Values are mean ± SD, (*n* = 6).

*P ≤ 0.05 mean difference, compared to CCl_4_-treated rats.

**P ≤ 0.001 mean difference, compared to CCl_4_-treated rats.
